# A Comparison of Currently Approved Small Interfering RNA (siRNA) Medications to Alternative Treatments by Costs, Indications, and Medicaid Coverage

**DOI:** 10.3390/pharmacy12020058

**Published:** 2024-03-28

**Authors:** Inder Sehgal, Kevin Eells, Imani Hudson

**Affiliations:** 1 College of Medicine, Rocky Vista University, Ivins, UT 84738, USA; 2 College of Medicine, Rocky Vista University, Parker, CO 80112, USA; kevin.eells@co.rvu.edu; 3 College of Pharmacy and Pharmaceutical Sciences, Florida A&M University, Tallahassee, FL 32307, USA

**Keywords:** siRNA, inclisiran, vutrisiran, patisiran, givosiran, lumasiran, Medicaid

## Abstract

Small interfering RNA (siRNA)-based medications offer the ability to target previously undruggable targets and have now received FDA approval in five instances for orphan or uncommon diseases. The current siRNA “-sirans” are directed towards hepatic molecular targets. Because they are not conventional drug formulae, their ultimate clinical success will require overcoming multiple barriers beyond their pharmacology. The minimal patient numbers leave fewer patients to bear the costs of R&D and manufacture; therefore, the cost of these drugs, questionable third-party reimbursement, and competition from other drug classes for the same low number of patients are impediments to patient access. The parenteral route of administration, as well as emerging safety restrictions, are also drawbacks to siRNA. With this review, we document currently approved siRNA drugs by condition, approval date, administration route and frequencies. We have estimated the available patient populations for siran therapies using the U.S. Medicaid and Medicare populations and sought to identify the frequency with which large Medicaid formularies list siRNA drugs. Current comparative costs between the siRNA drugs and alternatives have been presented, and the review summarizes current adverse events as reported to the FDA’s Adverse Event Reporting System. Our review and data indicate that sirans are extremely expensive and seldom recognized in posted Medicaid formularies. However, alternative treatments for these conditions are no less costly, usually do not have significantly different adverse events, and are often less convenient for the patient.

## 1. Introduction

In recent years, much attention in Medicaid pharmacy reimbursements has been focused on expensive medication such as antivirals used to treat hepatitis C (HCV) and newly approved biologics. These drugs generally have new mechanisms and formulations and may require special routes and timing of administration. A new medication category that also now fits this expense category is siRNA agents. Justification for their high costs arises from the orphan disease status of most of the current siRNA drug targets, but it is also due to the high costs of current competing or alternative treatments. If siRNAs are to become a standard modality of pharmacotherapy [1], costs will need to be paid by third parties such as Medicaid, which is a joint federal and state program and the single largest source of health coverage in the United States. However, coverage for expensive drugs such as siRNAs is seldom clearly defined on the websites of state Medicaid formularies [2], leaving open the question of coverage for patients with rare diseases.

Structurally, siRNAs are simply double-stranded RNA, one strand of which dissociates and anneals with a complimentary target mRNA, leading to degradation of the target and reducing translation and protein synthesis. This technology has enabled the elimination of what were previously “undruggable” targets [3], and they have merged as a class of medication therapies to suppress genes associated with orphan diseases and hypercholesterolemia [3]. At this point in time, six siRNA medications have been approved by the FDA. These medications are expensive when compared to traditional oral drugs, with costs per month ranging from USD 3500 for 6 months of inclisiran up to over USD 400,000 over three months for a loading dose of lumasiran (see data herein).

The first siRNA drug approval was for patisiran in August 2018 [3], and one new siRNA drug has been approved yearly since then [4]. There are upcoming approvals for fitusiran, teprasiran, and tivanisiran [4]. This technology has had to overcome pharmaceutical delivery obstacles, including rapid renal clearance, nuclease hydrolysis, and non-selective delivery [3]. These oligonucleotides easily cross into tissues with fenestrations; thus, they have been successfully targeted to liver conditions such as hepatic porphyria, hyperoxaluria, transthyretin, and hypercholesterolemia [3]. With chemical modifications such as phosphorothioate backbones and 2′-OH substitutions, approved siRNAs have increased half-life and reduced nuclease degradation. They are typically delivered using lipid nanoparticles with or without targeting ligands such as N-acetylgalactosamine sugars to target the asialoglycoprotein receptor on hepatocytes [5].

In this review, the currently FDA-approved siRNA drugs are briefly summarized according to their gene silencing mechanisms and the targeted disease. The patient populations in the U.S. with those siRNA-targeted diseases are estimated using Medicaid and Medicare data; then, medication costs are compared against the costs and administration routes of comparative therapies. Adverse effects associated with these therapies and with comparative therapies are compared using recent data from the FDA Adverse Events Reporting System (FAERS). State Medicaid formularies of the largest Medicaid population-serving states were researched to determine how frequently siRNA drugs are listed in comparison with alternatives.

## 2. siRNA Approved Medication Targets and Administration

The first FDA-approved siRNA was patisiran, sold as Onpattro, in August of 2018 [3,5] (Table 1). Patisiran is targeted to inhibit transthyretin for the treatment of hereditary transthyretin amyloidosis (ATTR) polyneuropathy and is formulated as a liposome. The manufacturer, Alnylam Pharmaceuticals, has also filed a supplemental new drug application (NDA) for patisiran for the treatment of ATTR cardiomyopathy [6]. The targeted RNA is the transcript for transthyretin (TTR), a 127-amino-acid protein that transports both thyroxine (T4) and retinol-binding protein bound to retinol. Patients with transthyretin amyloidosis hold a mutation in the *TTR* gene that expresses a misfolded protein; the aberrant protein leads to amyloid fibrils, which lead to both peripheral neuropathy and cardiomyopathy [7]. Alternative existing medication therapies for ATTR are tafamidis (Vyndaqel^®^, Vyndamax^®^), diflunisal (Dolobid^®^), and Inotersen (Tegsedi^®^). Tafamidis and diflunisal are small molecules that stabilize TTR within the homotetramer to prevent destabilization and dissociation into amyloid fibrils, therefore preventing the progression of TTR amyloidosis. Tafamidis is an analog of the non-steroidal anti-inflammatory drug diflunisal without nonsteroidal anti-inflammatory drug (NSAID) properties and was the first specific TTR-stabilizer to be approved by the FDA (May 2019) for the treatment of cardiomyopathy of wild-type or hereditary transthyretin-mediated amyloidosis (ATTR-CM) [7]. Diflunisal is an NSAID, used off-label [8]. Inotersen is an antisense phosphorothioate-modified oligonucleotide, approved in October 2018 [9], that targets and prevents the translation of transthyretin mRNA; it causes degradation of both the mutant and wild-type transthyretin mRNAs. This mechanism, while like siRNA in that both utilize sequence-specific suppression of gene expression, is different because siRNA use double-stranded RNA, while oligonucleotides use single-stranded DNA. Both lead to enzymatic cleavage of the target mRNA, albeit through differing nucleases [10]. Patisiran is given by intravenous infusion once every three weeks; inotersen can be self-administered once weekly, while both tafamidis and diflunisal are oral drugs. Both patisiran and inotersen may cause allergic-type reactions and vitamin A deficiency [11,12]. Because inotersen carries a black box warning for thrombocytopenia (low platelet counts) and kidney damage, it requires ongoing monitoring and is available only through a restricted distribution program under a risk evaluation and mitigation strategy (REMS). Patisiran does not require ongoing monitoring but needs to be administered by a healthcare professional.

The next siRNA drug approval was in November 2019 for givosiran, which was approved to treat acute hepatic porphyria [13] (Table 1). Givosiran inhibits hepatic delta-aminolevulinate synthase 1 (ALAS1) by reducing the levels of mRNA for ALAS1. Acute hepatic porphyrias (AHP) are a group of rare diseases that are characterized by episodes of acute neurovisceral pain secondary to excess accumulation of the neurotoxic porphyrin precursor delta-aminolevulinic acid (ALA). Acute hepatic porphyrias are the most common acute porphyrias and are the second most common porphyrias worldwide [13]. Patients with acute hepatic porphyria have a partial deficiency of the enzyme porphobilinogen (PBG) deaminase. When heme synthesis is stimulated in these patients, the PBG deaminase deficiency impairs full heme synthesis, and the lack of heme’s negative feedback induces the gene encoding delta-aminolevulinate synthase 1 (ALAS1). ALAS1 overproduces its product, ALA, as well as porphobilinogen and porphyrins in the liver. These porphyrin precursors are neurotoxic, and porphyrins absorb light, resulting in dermatologic toxicity [13]. Neurologic attacks occur acutely and may become chronic. The only definitive treatment for acute attacks is intravenous heme infusion [13], while liver transplantation is the long-term treatment for hepatic AHPs. Transplantation, however, does not affect neurologic heme synthesis deficiency. Hemin is available in the United States as a lyophilized hematin preparation (Panhematin). When infused intravenously, it is taken up primarily by hepatocytes, where it reconstitutes the heme pool; the resulting negative feedback represses ALAS1 synthesis. Givosiran is now the sole long-term medical therapy for hepatic porphyria. It is administered monthly via subcutaneous injection to treat recurrent acute attacks. The standard regimen for treatment is 3–4 mg/kg daily for 4 days [14]. For heme infusion, the standard regimen for treatment of acute attacks is 3–4 mg/kg daily for 4 days. 

Lumasiran (Oxlumo) was the third siRNA approval (approved in November 2020) to treat hyperoxaluria (Table 1). Primary hyperoxaluria results from a defective metabolic cascade beginning with reduced or absent alanine: glyoxylate aminotransferase activity. This hepatic enzyme normally uses glyoxylate and alanine as substrates and produces pyruvate and glycine. Reduced/absent enzyme activity leads to buildup of the glyoxylate substrate, which is converted into oxalate and transported to the kidney [15]. Oxalate forms a salt as calcium oxalate, the main component of renal and cystic stones. Chronic damage to the kidneys can lead to end-stage renal disease. As renal function declines, reduced oxalate excretion leads to hyperoxalemia and systemic oxalosis due to oxalate deposition in other tissues [16]. Lumasiran reduces the synthesis of glyoxylate by triggering the degradation of the mRNA from the hydroxyacid oxidase 1 gene (*HAO1*); this mRNA encodes the glycolate oxidase enzyme that synthesizes glyoxylate. By reducing hepatic glyoxylate production, oxalate levels decrease in the plasma and urine [15]. Lumasiran is administered subcutaneously monthly for the first 3 months, then every three months.

Prior to lumasiran, therapeutic small molecular drugs can only manage symptoms of hyperoxaluria; they cannot cure the disease. For patients with either primary or secondary hyperoxaluria, these options include oral doses of potassium citrate or the combination of orthophosphate and magnesium to prevent calcium oxalate crystals from forming. Vitamin B-6 (pyridoxine) and thiazide diuretics can reduce oxalate levels for a specific subtype of PH. Organ transplant of either or both the kidney and liver are another treatment option [16]. 

Inclisiran was the fourth siRNA approved, and it was approved in December 2021 to treat clinical atherosclerotic cardiovascular disease or heterozygous familial hypercholesterolemia (HeFH) in patients who require additional lowering of LDL-c [17] (Table 1). Familial hypercholesterolemia is an autosomal dominant condition resulting from mutations in the LDL-receptor gene that lead to reduced LDL clearance [18]. Inclisiran targets mRNA that would encode proprotein convertase subtilisin K9, the enzyme that degrades LDL receptors; thus, inclisiran indirectly increases LDL receptors [19]. Hypercholesterolemia is initially treated using statins as the drug class of choice, and familial hypercholesterolemia is typically treated using “high-intensity” statin therapy. Since many familial hypercholesterolemia patients do not reach target LDL-C levels, other medication options are the addition of the cholesterol absorption inhibitor ezetimibe, bile acid sequestrants, and PCSK9-targeting monoclonal antibodies [20]. Pooled trial data from three phase II trials indicate that inclisiran is effective in lowering LDL-C levels in in heterozygous familial hypercholesterolemia high-risk patients in whom LDL-C is not reduced to acceptable levels with a statin (with or without ezetimibe) alone [21]. 

The PCSK9 antibodies evolocumab and alirocumab target the PCSK9 enzyme in plasma and were the first biologics approved for heterozygous familial hypercholesterolemia (HeFH), as well as for resistant hyperlipidemias [22]. These fully humanized monoclonal antibodies bind the free PCSK9 protein, obstructing LDL receptor degradation and thereby increasing LDL clearance. LDL apheresis, a physical method of purging the blood of LDL, is an option for those for whom heterozygous familial hypercholesterolemia is not managed using pharmacotherapies [23]. This extracorporeal treatment for LDL cholesterol elimination can reduce cholesterol by 50–60% but necessitates retreatment every 7–14 days. High costs are a disadvantage of apheresis [20]. Inclisiran is dosed at 284 mg in 1.5 mL initially administered as a single subcutaneous injection, then after 3 months and every 6 months thereafter. Inclisiran can be stored at room temperature.

Vutrisiran, approved in June 2022, is like patisiran for the therapy of transthyretin amyloidosis peripheral neuropathy and targeted to the transthyretin mRNA (Table 1). It differs from patisiran in that the administration is subcutaneous and every 3 months instead of via IV infusion every 3 weeks [24].

Nedosiran, newly approved in September 2023, is administered subcutaneously on a monthly basis and targets hepatic lactate dehydrogenase (Table 1), which is involved in primary hyperoxaluria type 1 [25,26]. This agent overlaps with lumasiran in indication, patient population, and therapeutic alternatives. Accurate costs, adverse event report numbers, and state Medicaid approvals will likely emerge later in 2024; therefore, these data are not part of this review.

**Table 1:** Summary of siRNA Drugs by condition, approval date, administration route, and frequency.

Medication Generic (Brand)	Therapeutic Condition	Therapeutic Target	Approval	Administration	Frequency
Patisiran (Onpattro)	Hereditary transthyretin amyloidosis polyneuropathy	Transthyretin	August 2018	Intravenous Infusion	Every 3 weeks [27]
Givosiran (Givlaari)	Acute hepatic porphyria	Delta-aminolevulinate synthase 1	November 2019	S.C.	1 month [28]
Lumasiran (Oxlumo)	Hyperoxaluria	Glycolate oxidase enzyme	November 2020	S.C.	Loading: monthly (3 months); Maintenance: every 3 Months [29]
Inclisiran (Leqvio)	Atherosclerotic cardiovascular disease or heterozygous familial hypercholesterolemia	Proprotein convertase subtilisin K9	December 2021	S.C.	Loading: first dose, second dose at 3 months, then every 6 months [30]
Vutrisiran (Amvuttra)	Hereditary transthyretin amyloidosis polyneuropathy	Transthyretin	June 2022	S.C.	3 months [24]
Nedosiran (Rivfloza)	Primary hyperoxaluria type 1	Hepatic lactate dehydrogenase	September 2023	S.C.	Monthly [25,26]

## 3. Population Numbers with siRNA Drug Condition

siRNA medications have thus far been approved for rare conditions except for the indication of hypercholesterolemia. A rare condition defined by the orphan drug act is “a disease or condition that affects less than 200,000 people in the United States” [31]. To estimate the prevalence of patients in the U.S. with siRNA-drug-treatable conditions, we determined the prevalence of these conditions among those enrolled in either Medicaid or Medicare [32,33]. The combined total populations receiving Medicaid and Medicare is over 150 million people, representing approximately 45% of the 2022 population. For every 1 million Medicaid and Medicare enrollees, 6.5 people were diagnosed with hereditary transthyretin-mediated amyloidosis, 8.5 were diagnosed with acute intermittent (hepatic) porphyria, 3.7 with hyperoxaluria, and 7017 with hypercholesterolemia or heterozygous familial hypercholesterolemia (Table 2). These low patient numbers contribute to the high drug expenses since the development and production costs are divided among only a few patients. 

## 4. Comparative Costs of siRNA Therapies

Although the siRNA medications are far more expensive per dose than small-molecule drugs for common conditions, the costs of siRNAs are similar to existing alternative therapies for the orphan diseases they target. Cost comparison estimates between the approved siRNA drugs and comparative therapies are shown in Table 3. To calculate the costs of medication costs dosed per kilogram of body weight, calculations are made for a 70 kg individual. For comparison purposes, costs are also expressed on an annualized basis. These data show that for transthyretin amyloidosis, patisiran costs USD 375,561 annually. This compares with USD 263,844 for a year of tafamidis capsules, USD 389,376 for inotersen antisense oligonucleotides, and USD 502,744 annually for vutrisiran. Cost is one factor and needs to be balanced against efficacy, ease of administration, and adverse effects. There are no head-to-head comparisons of patisiran and tafamidis; however, one indirect analysis suggested that patisiran has a greater treatment effect on neuropathy impairment Score—lower limbs and quality of life diabetic neuropathy than tafamidis in patients with hATTR amyloidosis with polyneuropathy [35].

Patisiran has also demonstrated greater treatment effects on neuropathy and quality of life (QOL) than inotersen in patients with hATTR amyloidosis with polyneuropathy [36]. Patisiran and vutrisiran have not been compared head-to-head and they are both manufactured by Alnylam Pharmaceuticals. Both target an mRNA sequence that is conserved in both wild-type and TTR variants. Compared with vutrisiran, patisiran is administered more frequently (every 3 weeks vs. every 3 months). The APOLLO clinical trial for patisiran concluded that it was well tolerated, with no increases in the frequency of adverse events compared to placebo by organ system; infusion-related reactions were mild in severity. The HELIOS-A clinical trial for vutrisiran reported adverse reactions of at least 5% for arthralgia and dyspnea and decreased vitamin A (7%); injection site reactions were mild and transient [37]. Thus, the cost of patisiran is comparable to other available hATTR therapies, and while vutrisian is more expensive on a monthly basis, the once-every-three-months dosing offers convenience to patients with decreased mobility due to ATTR. 

The costs to treat a 70 kg person with acute intermittent hepatic porphyria with subcutaneous givosiran is USD 484,188 annually. In comparison, hemin twice-weekly prophylaxis at 4 mg/kg is estimated to cost USD 889,075 per year, and the cost of a liver transplant is approximately USD 878,400 [38]. As stated above, hemin is administered IV and works to treat acute episodes or would need to be given chronically, twice-weekly, for prophylaxis. Liver transplantation would be curative for hepatic but not neurologic porphyrias. Therefore, the relative cost of givosiran is lower when compared with hemin administrations.

Lumasiran administered subcutaneously for hyperoxaluria for a 70 kg patient would cost USD 1,638,694 for the first year. The costs for pyridoxine, K-citrate, and other oral medications are under USD 120 annually, yet these are only symptomatic treatments. Renal transplant at approximately USD 414,800 [39] and liver transplant are the surgical alternatives if organs are available. Relative to these expenses, lumasiran, as the drug therapy, could exceed the costs of transplant over time but without surgical morbidities and immunosuppressive medications.

Inclisiran is the least costly siRNA approved. The average annual cost is similar to the cost for the anti-PCSK9 antibodies alirocumab and evolocumab (Table 3). The antibodies are administered every two weeks, while inclisiran offers the advantage of every-6-months dosing after a 3-month initiation dose.

## 5. Comparative Safety

The most current documentation of the adverse events reported for each of the siRNA drugs was obtained using the FDA’s Adverse Event Reporting System (FAERS) [50], and these data were compared against the adverse events (AEs) reported for major alternative therapies for the same time intervals in Table 4. These safety reports list the relative reporting of AEs by health professionals and consumers. Since its 2018 approval, patisiran’s most reported adverse event (AE) was death at 7.8%, and multiple other AEs associated with patisiran were spread amongst various reactions. Death was also the most frequent AE reported for tafamidis at 27.6%, while thrombocytopenia (23.7%) was most common for inotersen (deaths were reported at 2.7%). Vutrisiran is relatively new and has fewer reported AEs; death makes up 10.3% of the reports. Since the reports do not prove that a specific product caused the adverse event and do not show incidence or prevalence of an event, the difference in deaths between the three therapies cannot be causality linked; however, they point to the importance of monitoring for safety in patients treated with patisiran, vutrisiran, and tafamidis. 

The most frequent AE reported for givosirin is acute porphyria (32%) compared with phlebitis (9%) for hemin infusions, suggesting that some patients do not respond to siRNA therapy. Death reports for both therapies are low.

Lumasiran had no deaths reported since approval, and the most common reaction reported was lack of effect (FAERS lists reactions as reported by reporters and includes “lack of effect” and “device difficult to use” as reactions). The supplements pyridoxine and potassium caused a higher percentage of deaths, although these data are for all uses and not limited to hyperoxaluria. The most frequent AEs for pyridoxine and potassium are nausea and lack of effect, respectively.

Inclisiran has a relatively low percentage of death reports, as do the alternatives alirocumab and evolocumab. The most frequent AEs reported for all three injectables were related to the delivery rather than the mechanism (injection-site pain for inclisiran, and product delivery and device use difficulty for alirocumab and evolocumab).

## 6. Medicaid Coverage and Disease Incidence

The significant costs of siRNA medications, as with other costly new therapies, are a challenge for third party payers. State Medicaid programs, as the payer for citizens near the poverty limits, are burdened by the high costs of orphan therapies. No data so far have addressed formulary listings for siRNA drugs by Medicaid programs. To determine the extent of formulary listings for siRNA drugs, state formulary sites were accessed and searched for those Medicaid states with the largest populations of enrollees. Of the 16 states with a Medicaid/CHIP enrollment of 2 million or more, only Arizona, California, Massachusetts, and Washington listed any of the siRNA drugs in their formulary/preferred drug lists (Table 5). In these cases, prior authorization was required. The other state Medicaid websites did not list any siRNA drugs, although individual states may elect to cover siRNA drugs on a case-by-case basis based on requests by the physician or provider. Drug formularies shape provider prescription options, and since most states fail to list siRNA drugs, these drugs may not be top options for Medicaid patients with orphan diseases. The National Average Drug Acquisition Cost (NADAC) 2023 comparison lists no rates for any siRNA drugs [52].

## 7. Conclusions

siRNA drug therapies have been available for fewer than 5 years. Their structures are double-stranded RNAs with one strand complimentary to a target mRNA that encodes a protein associated with disease. The degradation of the target mRNA via siRNA-induced endonuclease activity reduces translation and therefore target protein levels. By functioning at the RNA level instead of at the protein level, siRNA can eliminate targets that are not reachable as proteins. Since the mRNA sequences for targets are often known, siRNA oligonucleotides can be synthesized with lower research and development costs, and this, in part, explains their most common drug approvals for rare diseases. However, siRNA therapeutics still carry high costs due to the additional technologies needs for drug targeting and delivery, nucleotide stability, non-oral administration routes, packaging, and low patient numbers for reimbursement. However, comparative data in this review indicate that the high costs of siRNA drugs are comparable to alternative and competing therapies that have similar efficacies and duration of effects.

Safety data present on the FAERS database involve relatively few patients because of the rareness of the diseases and newness of the siRNAs. The deaths reported with use of patisiran point to the need for continued monitoring; however, death is also reported for the transthyretin amyloidosis polyneuropathy alternative tafamidis. The other approved siRNA drugs have issues including a lack of efficacy, delivery complaints, and worsening of the disease condition. Data presented here also found that the largest state Medicaid systems seldom recognize any of the approved siRNA agents in their posted formulary or preferred drug lists. However, since Medicaid formularies are open in the sense that they can provide nearly all prescribed drugs made by manufacturers, states may approve these and other expensive agents after a provider provides sufficient prior authorization [69]. If these and other costly agents for rare conditions are to continue into the market, they must recoup research, development, and production costs; therefore, both state Medicaid and federal Medicare programs will need to evolve a means for reimbursement. These data show that importantly, the alternative treatments for these conditions are not less costly, usually do not have significantly different adverse events, and are often less convenient for the patient.

## Figures and Tables

**Table 2:** The 2022 U.S. Medicaid and Medicare population numbers with siRNA drug conditions.

Chronic Conditions Data Warehouse [33,34]	Diagnosis Code	Estimation of Medicaid and Medicare Population Numbers †
Hereditary transthyretin-mediated amyloidosis	E85.82	98 Maid; 875 Mcare outpatient
Acute intermittent (hepatic) porphyria	E80.21	740 Maid; 540 Mcare outpatient
Primary hyperoxaluria	E72.53	374 Maid; 1594 Mcare outpatient
Hypercholesterolemia	E78.00	1,017,579 Maid; 2,306,897 Mcare outpatient
Heterozygous familial hypercholesterolemia	E78.01	37,782 Maid; 70,423 Mcare outpatient

† Medicaid population estimates are extrapolated from one year of Medicaid data. Approximately 85,280,085 individuals were enrolled in Medicaid. Approximately 65,103,807 people are enrolled in Medicare.

**Table 3:** Comparison of siRNA vs. alternative therapies by costs and route.

siRNA/Route of Administration	NDC	Cost/Year	Comparative Rx	Cost/Year Unless as Noted	Route of Comparative	Removes Aberrant Protein
Patisiran /IV	71336-1000-01	USD 375,561 ^a^ [35]	Tafamidis (non-NSAID diflunisal analog)	USD 263,844 ^b^ [36]	Oral	No
			Inotersen (antisense oligonucleotide)	USD 389,376 ^c^ [37,38]	S.C.	Yes
Givosiran/S.C.	71336-1001-01	USD 484,188 ^d^ [39]	Hemin twice week prophylaxis	USD 889,075 ^e^ [40]	IV	No
			Liver transplantation	USD 878,000 lifetime [41]	Surgical	Yes hepatic; No neurologic
Lumasiran/S.C.	71336-1003-01	USD 1,638,694 ^f^ [6,42]	Pyridoxine	Under USD 120 [43]	Oral	No
			Potassium citrate solution	Under USD 120 [44]	Oral	No
			Kidney transplant	Renal transplant USD 414,800 lifetime [45]	Surgical	No
Vutrisiran/S.C.	71336-1003-1	USD 502,744 ^g^ [46]	As for Patisiran; Patisiran is an alternative	As for Patisiran		Yes
Inclisiran/S.C.	00078-1000-06	USD 6948 ^h^ [47]	Alirocumab	USD 6539 ^i^ [48]	S.C.	No
			Evolocumab	USD 7306 ^j^ [49]	S.C.	No

^a^ USD 10,313 for a supply of 5 milliliters/10 mg vial, @ 0.3 mg/kg = 21 mg for 70 kg patient; therefore USD 21,657 every 3 weeks annualized to 1 year; ^b^ USD 21,987 for 30 × 61 mg capsules (1 month) annualized; ^c^ USD 284 mg/week.at USD 7488/week annualized; ^d^ USD 43,577 for 189 mg given at 2.5 mg/kg BWt/month × 70 kg = 175 mg/month = USD 40,349/month annualized; ^e^ USD 10,686/350 mg powder, 4 mg/kg × 70 kg = 280 mg × 2 days/week = 560 mg/week = USD 17,097 annualized; ^f^ USD 61,451 for 94.5 mg, 3 mg/kg monthly for first 3 months × 70 kg = 210 mg/month loading = USD 136,558/month × 3 month s = USD 409,674; then, 210 mg/every 3 months = USD 1,229,020 for 9 months = USD 1,638,694 annualized; ^g^ USD 125,686 for 25 mg given every 3 months annualized; ^h^ USD 579/month annualized; ^i^ USD 503/2 × single-dose pens, one pen every 2 weeks, annualized. ^j^ USD 562/2 × single-dose pens; one pen every 2 weeks, annualized.

**Table 4:** siRNA and alternative drug adverse events reported to FAERS.

FDA Adverse Events Reporting System (FAERS) Public Dashboard [51]	Number of Reported AE Since Approval Year	FAERS Deaths	Most Common Reaction Category	Percent of Adverse Event Cases with Most Common Reaction
Patisiran (2018–2023)	2024	157	Death	7.8%
Tafamidis (2018–2023)	6787	1874	Death	27.6%
Inotersen (2019–2023)	1004	27	Platelet count decreased	23.7%
Givosiran (2019–2023)	487	11	Acute porphyria	32.7%
Hemin (2019–2023)	118	6	Phlebitis	7.6%
Lumasiran (2020–2023)	53	0	Drug ineffective	13.2%
Pyridoxine (2020–2023)	784	174	Nausea	17.9%
Potassium citrate (2020–2023)	235	8	Drug ineffective	17.5%
Inclisiran (2021–2023)	1426	46	Injection site pain	12.8%
Alirocumab (2021–2023)	3413	143	Product delivery mechanism issue	8.0%
Evolocumab (2021–2023)	27,784	423	Device difficult to use	32.2%
Vutrisiran (2022–2023)	87	11	Death	10.3%

**Table 5:** State Medicaid formulary listing of siRNA Drugs.

State	Number of Medicaid/CHIP Enrolled (×1,000,000)	Number of Medicaid/Enrolled (×1,000,000)	Medicaid Web Site siRNA Drugs in Formulary or Preferred Drugs Lists
California	14.0	12.7	Inclisiran, Vutrisiran, Patisiran, Givosiran, Lumasiran [53]
New York	7.3	6.8	None [54]
Texas	5.7	5.3	None [55]
Florida	4.9	4.8	None [56]
Ohio	3.3	3.1	None [57]
Michigan	3.0	2.1	None [58]
Illinois	3.8	3.5	None [59]
Pennsylvania	3.6	3.4	None [60]
Arizona	2.3	2.1	Patisiran, Givosiran, Lumasiran, Vutrisiran, Inclisiran, [61]
Georgia	2.5	2.1	None [62]
New Jersey	2.2	1.9	None [63]
North Carolina	2.3	2.0	None [64]
Indiana	2.0	1.9	None [65]
Virginia	2.0	1.8	None [66]
Massachusetts	2.0	1.8	Vutrisiran, Patisiran, Givosiran, Lumasiran [67]
Washington	2.1	2.1	Vutrisiran, Givosiran, Lumasiran, Patisiran [68]

## Data Availability

The data presented in this study are available in the article.

## Abbreviations

AE	Adverse event
AHP	Acute hepatic porphyria
ATTR	Transthyretin Amyloidosis
ALA	Aminolevulinic acid
ALAS1	Delta-aminolevulinate synthase 1
FAERS	FDA’s Adverse Event Reporting System
FDA	Food and Drug Administration
HCV	Hepatitis C virus
hATTR	Hereditary transthyretin amyloidosis
IV	Intravenous
siRNA	Small interfering RNA
LDHA	Lactate dehydrogenase
LDL-c	Low-density lipoprotein cholesterol
Maid	Medicaid
Mcare	Medicare
NADAC	National Average Drug Acquisition Cost
NDA	New drug application
PBG	Porphobilinogen deaminase
PCSK9	Proprotein convertase subtilisin/kexin type 9
REMS	Risk evaluation and mitigation strategy
TTR	Transthyretin
